# Overexpression of *TaLEA* Gene from *Tamarix androssowii* Improves Salt and Drought Tolerance in Transgenic Poplar (*Populus simonii × P. nigra*)

**DOI:** 10.1371/journal.pone.0067462

**Published:** 2013-06-28

**Authors:** Weidong Gao, Shuang Bai, Qingmei Li, Caiqiu Gao, Guifeng Liu, Guangde Li, Feili Tan

**Affiliations:** 1 State Key Laboratory of Tree Genetics and Breeding, Research Institute of Forestry, Chinese Academy of Forestry, Beijing, China; 2 State Key Laboratory of Tree Genetics and Breeding, Northeast Forestry University, Harbin, China; 3 School of Agroforestry & Medicine, the Open University of China, Beijing, China; 4 School of Life Science & Technology, Zhanjiang Normal University, Zhanjiang, China; 5 Engineering & Garden Department of Beijing Ba Da Chu Park, Beijing, China; TGen, United States of America

## Abstract

Late embryogenesis abundant (LEA) genes were confirmed to confer resistance to drought and water deficiency. An *LEA* gene from *Tamarix* *androssowii* (named *TaLEA*) was transformed into Xiaohei poplar ( *Populus* *simonii* *× P. nigra*) via *Agrobacterium* . Twenty-five independent transgenic lines were obtained that were resistant to kanamycin, and 11 transgenic lines were randomly selected for further analysis. The polymerase chain reaction (PCR) and ribonucleic acid (RNA) gel blot indicated that the *TaLEA* gene had been integrated into the poplar genome. The height growth rate, malondialdehyde (MDA) content, relative electrolyte leakage and damages due to salt or drought to transgenic and non-transgenic plants were compared under salt and drought stress conditions. The results showed that the constitutive expression of the *TaLEA* gene in transgenic poplars could induce an increase in height growth rate and a decrease in number and severity of wilted leaves under the salt and drought stresses. The MDA content and relative electrolyte leakage in transgenic lines under salt and drought stresses were significantly lower compared to those in non-transgenic plants, indicating that the *TaLEA* gene may enhance salt and drought tolerance by protecting cell membranes from damage. Moreover, amongst the lines analyzed for stress tolerance, the transgenic line 11 (T11) showed the highest tolerance levels under both salinity and drought stress conditions. These results indicated that the *TaLEA* gene could be a salt and drought tolerance candidate gene and could confer a broad spectrum of tolerance under abiotic stresses in poplars.

## Introduction

Various abiotic stresses, especially drought and salinity, have a substantial impact on plant growth and development. To ensure both their own survival and the prosperity of their offspring, plants have developed a range of strategies, including regulation of gene expression, in order to cope with adverse conditions through various physiological adaptations. Late embryogenesis abundant (LEA) is one of the most important stress-associated gene families. The *LEA* proteins were first identified and characterized in cotton (*Gossypium hirsutum*) [1], and are highly expressed during the later stages of embryogenesis in higher plants. To date, many *LEA* genes have been identified in different plant species, and many of the *LEA* genes have been demonstrated to be associated with tolerance against water deficiency, salt and osmotic and freezing stresses [2–7].

The overexpression of *LEA* genes can improve the salt and drought stress tolerance of transgenic plants. Overexpression of Rab16A (Group 2 *LEA*) in indica variety of rice conferred increased salt tolerance [8]. Overexpression of AtLEA3-3 in *Arabidopsis* confers salt and osmotic stress tolerance that is characterized during germination and early seedling establishment [9]. Muñoz-Mayor et al. [10] also identified that the plants overexpressing tas14 gene achieved improvement in long-term drought and salinity tolerance without affecting plant growth under non-stress conditions.

*T* *. androssowii* possesses strong resistance to abiotic stresses, including salinity, drought and high temperature, which make it an ideal species to investigate the mechanism of stress tolerance in tree and to clone the stress tolerance gene. Wang et al. [11] confirmed that a *TaLEA* gene from *T* *. androssowii* could improve drought tolerance in transgenic tobacco (*Nicotiana tabacum*) plants. However, forest trees and herbaceous plants may possess different tolerance mechanisms. To study how the *TaLEA* gene improves tolerance in a tree species, it was transformed into Xiaohei poplars ( *P. simonii* *× P. nigra*) via *Agrobacterium* . Eleven transgenic lines were selected for salt and drought stress analysis. The malondialdehyde (MDA) content, relative electrolyte leakage and the relative plant growth rate were investigated in transgenic and non-transgenic (wild type) Xiaohei poplars. The results showed that these physiological parameters were significantly affected by salt and drought stresses, and that the transgenic poplars exhibited a higher stress tolerance than non-transgenic plants. The results suggest that the *TaLEA* gene is a stress tolerance gene and may be responsible for enhancing salt and drought tolerance in forest trees.

## Results

### Confirmation of transgenic plants by polymerase chain reaction (PCR) and ribonucleic acid (RNA) gel blot

**Figure 1:**
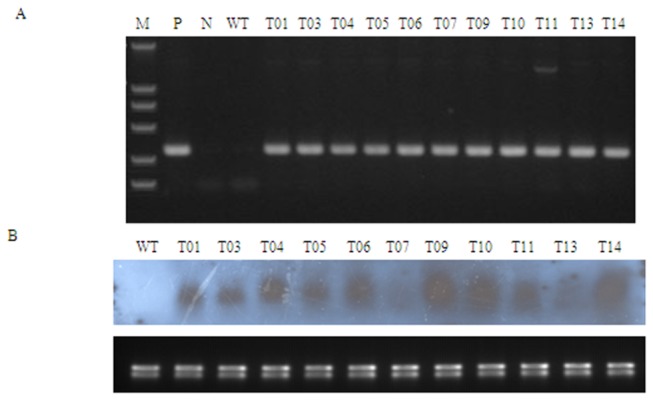
RT-PCR and RNA gel electrophoresis showing overexpression of *TaLEA* in poplars. A: Detection of the transgene from kanamycin-resistant lines by RT-PCR; M: DNA molecular weight marker (DL2000), P: positive control (pROKII-*TaLEA*); N: negative control using water as PCR template; WT: negative control using DNA from WT plants as PCR template; T01-T14: independently transformed poplar lines. B: Analysis of transgene expression in transgenic lines by RNA gel blot analysis; WT: wild type plant; T01-T14: independently transformed poplar lines.

Xiaohei poplar explants infected with *Agrobacterium* and containing the *TaLEA* gene were selectively cultured on kanamycin medium. Twenty-five independent transgenic lines were obtained with tolerance to kanamycin and 11 transgenic lines were randomly selected for further analysis. All 11 lines produced the expected amplification product of 312 bp determined by PCR using the *TaLEA* specific primers (Figure 1A), indicating that they were true transformants. The results of RNA gel blot further showed that *TaLEA* was expressed in 11 transgenic lines (Figure 1B), suggesting that the *TaLEA* gene had been integrated into the poplar genome and stabilized expression. However, there was great variability on the expression levels among the lines. The expression level of lines 6, 9, 10 and 14 was high. On the other hand, the lines 7 and 13 showed relatively low expression.

### Comparison of malondialdehyde (MDA) content among transgenic lines and non-transgenic plants

**Figure 2:**
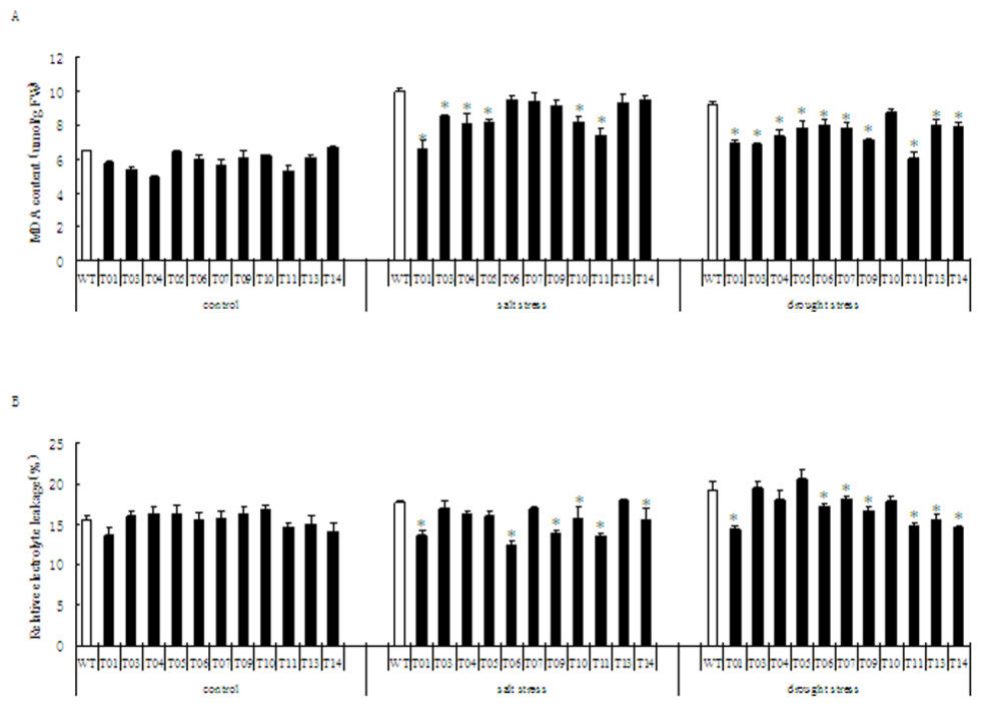
Effect of *TaLEA* transgene expression on malondialdehyde (MDA) content and electrolyte leakage of Poplars. Histograms showing A: MDA content comparison. B: Relative electrolyte leakage comparison; control: normal condition; salt stress: 200 mM NaCl treatment for 6 d; drought stress: withholding water for 7 d between WT and *TaLEA*-transformed poplars. At least five plants from each line were used for biological repeats in each experiment. Values are means ± S.D. and the level of significance was set at *P* < 0.05. The stars above the bars indicate significant differences (*P* < 0.05) between the transgenic lines compared with WT under the test conditions.

Since MDA is an end-product of free radical chain reactions and lipid peroxidation in biomembranes, MDA content can represent the extent of lipid peroxidation and membrane injury that has occurred. Therefore, the MDA contents of 11 transgenic lines and non-transgenic plants were measured before and after a 200 mM NaCl treatment or drought stress. The results showed that there were no significant differences in MDA contents in transgenic lines and non-transgenic plants before stress. The MDA contents were highly increased after salt and drought stresses in both transgenic lines and non-transgenic plants. However, the MDA contents in the transgenic lines, especially the lines 1 and 11, were significantly (*P* < 0.05) lower than those in the non-transgenic plants (Figure 2A). The MDA content was increased by 54.1% in non-transgenic plants and by 13% in transgenic lines on an average after the salt stress. Similarly, differences in MDA contents were observed between transgenic lines and non-transgenic plants after the drought stress. The transgenic line 11 showed the lowest MDA content. These results implicated that salt and drought stresses gradually injured plant membranes through lipid peroxidation, while the product of the exogenous *TaLEA* gene played a vital role in a protective antioxidation system.

### Comparison of relative electrolyte leakage between transgenic and non-transgenic poplars

No significant difference was observed between transgenic and non-transgenic poplars in the relative electrolyte leakage before stress. However, the relative electrolyte leakage of these plant lines was notably different after salt and drought stresses. With the exception of the line T13, the relative electrolyte leakage in all transgenic lines was lower than those of the non-transgenic plants after the salt stress (Figure 2B). The lowest relative electrolyte leakage was found in lines T6 and T11, where it was 30.5% and 23.6% lower than that of the non-transgenic plants, respectively. For the drought stress, analysis of variance also showed notable differences in the relative electrolyte leakage between most transgenic lines and non-transgenic plants.

### Stress damage analysis

**Figure 3:**
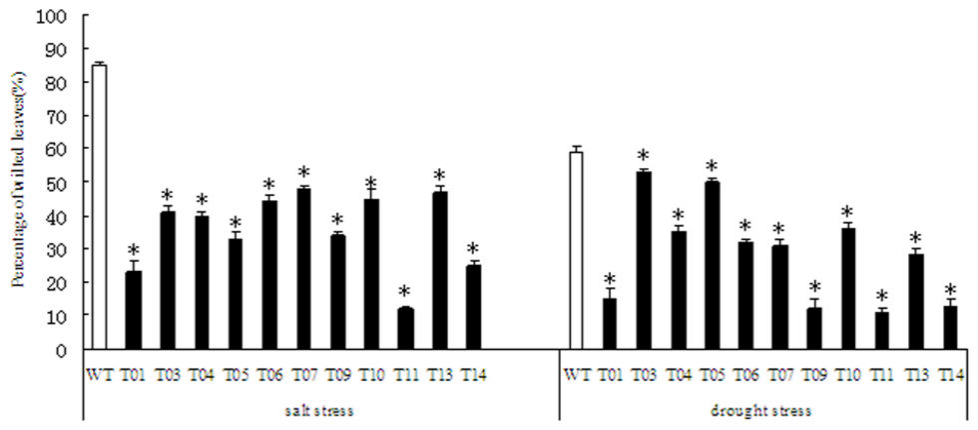
The effects of salt and drought stress on leaf wilting of transgenic and non-transgenic poplar plants. Histograms showing the percentage of wilted leaves in WT and *TaLEA*-transformed poplars under salt and drought stress. At least five plants from each line were used for biological repeats in each experiment. Values are means ± S.D. and the level of significance was set at *P* < 0.05. The stars above the bars indicate significant differences (*P* < 0.05) between the transgenic lines and WT under the test conditions.

**Figure 4:**
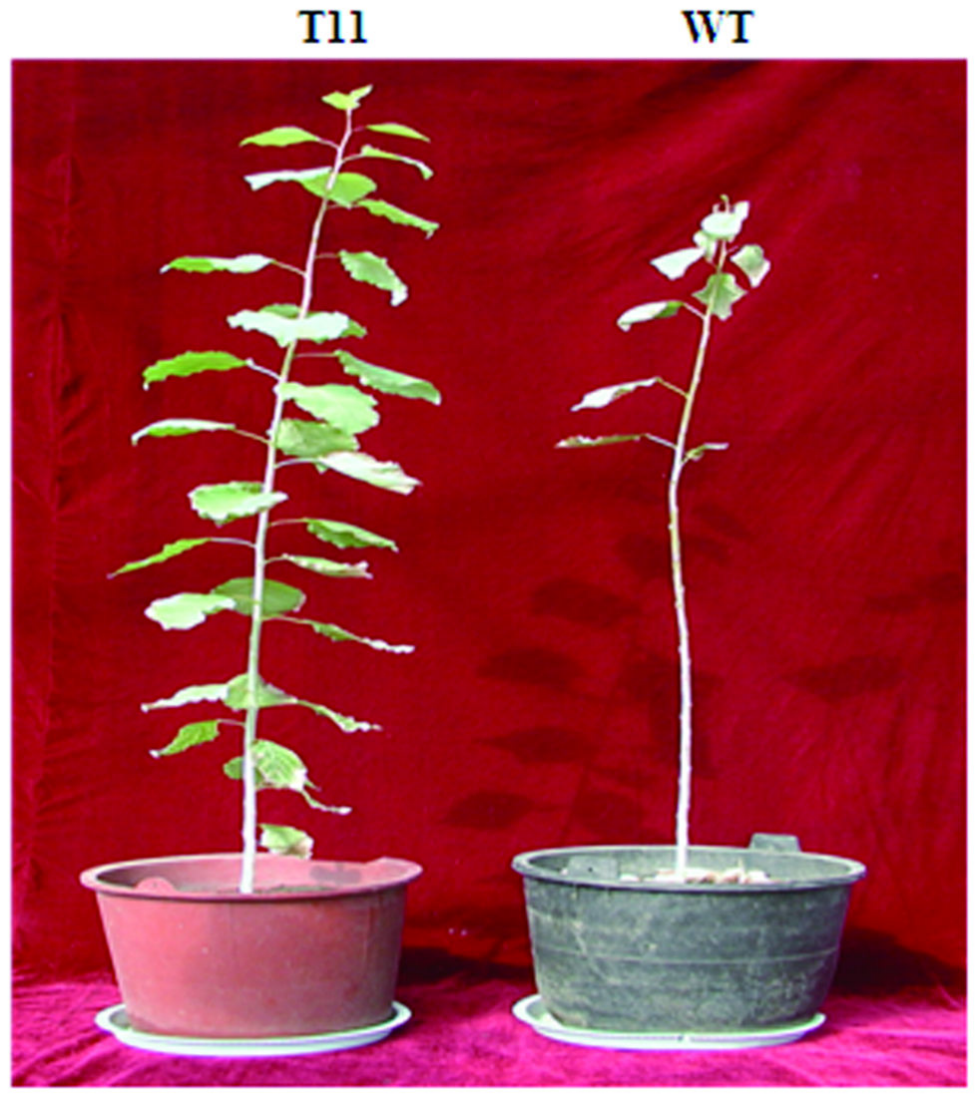
Appearances of transgenic lines and non-transgenic plants after 30 days of salt stress. A photograph showing *TaLEA*-transformed and WT poplar plants after 30 days of salt stress. T11: transgenic line 11; WT: wild type.

After the plants were watered with 200 mM NaCl solution for 30 days, 85% of the non-transgenic plant leaves wilted, while the percentage of wilted leaves among the transgenic lines ranged from 12% to 48% (Figure 3). Of all transgenic lines, the line T11 showed the least damage. However, the non-transgenic plants showed only very few normal leaves (Figure 4). The number of wilted leaves was lower in transgenic lines than in the non-transgenic plants after deficiency of water. These results indicated that overexpression of *TaLEA* enhanced salt and drought tolerance in transgenic poplars.

### Comparison of relative rate of height growth between transgenic and non-transgenic poplars

**Figure 5:**
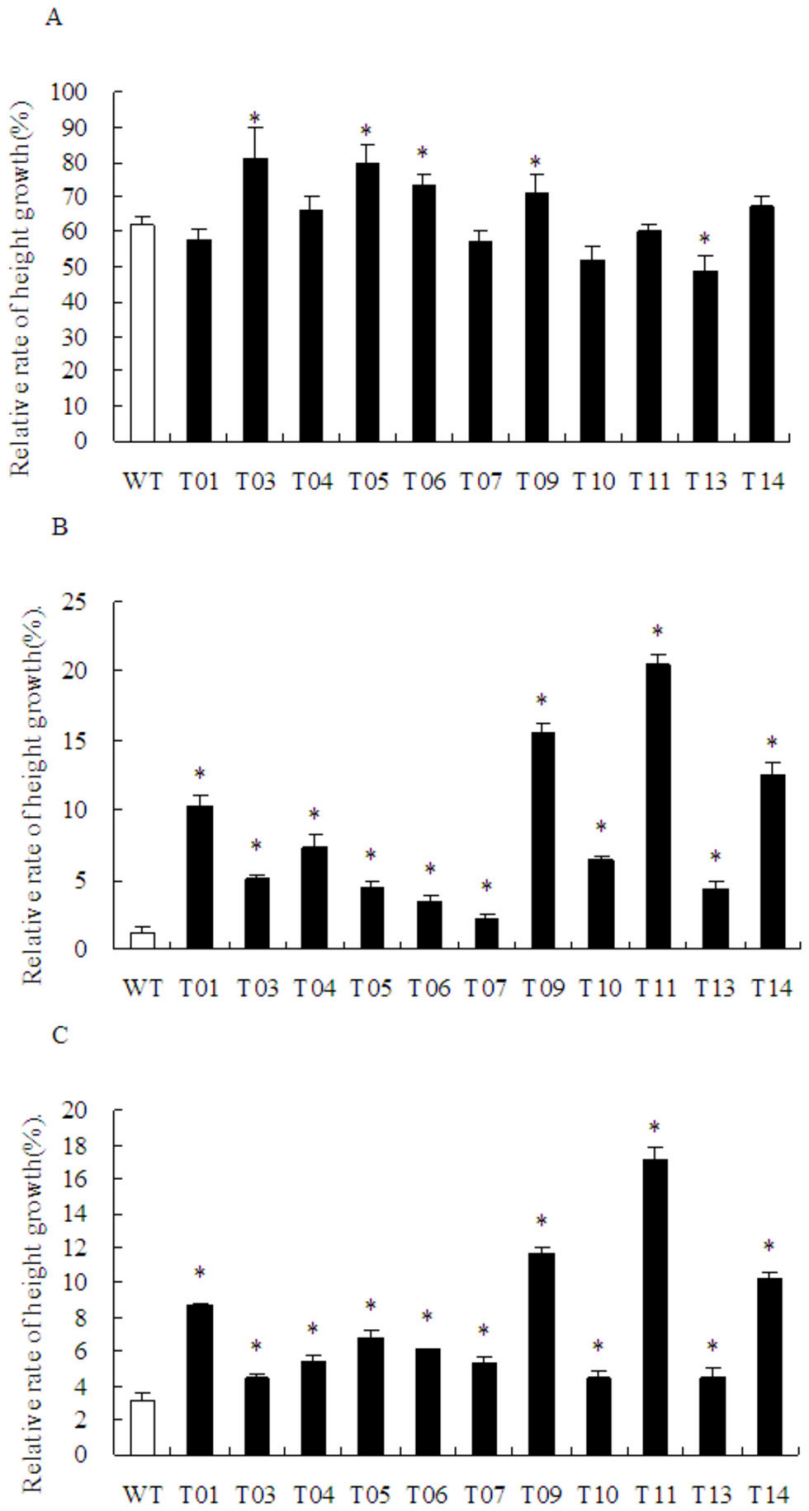
Effects of stress on the relative rate of growth of WT and transgenic plants. Histograms showing a comparison in the rate of growth between WT and *TaLEA*-transformed poplars. A: Control condition; B: 200 mM NaCl treatment for 6 d; C: Withholding water for 7 d. At least five plants from each line were used for biological repeats in each experiment. Values are means ± S.D. and the level of significance was set at *P* < 0.05. The stars above the bars indicate significant differences (*P* < 0.05) between the transgenic lines compared with WT.

The heights of transgenic lines and non-transgenic plants were measured before and 30 days after introducing stress by adding NaCl solution. Under constant stress-free conditions, not all transgenic lines grew faster than non-transgenic plants. However, there was a significant difference between the transgenic lines and non-transgenic plants after the NaCl stress. The relative growth rates of the transgenic lines ranged from 2.2% to 20.5%, which were much higher than that of non-transgenic plants (1.2%). Especially, the T11 line showed the highest (20.5%) relative growth rates (Figure 5). These results were consistent with the results of damage analysis. The relative height growth rate response to water deficit stress was similar to the NaCl stress.

## Discussion

Up to date, there have been many reports published on development of transgenic plants with improved drought and salt stress tolerance by overexpression of stress-related genes in herbs [12–17]. However, relatively few studies have been carried out on trees [18–20]. There are many differences between trees and herbaceous plants, such as structure, growth, development and physiology in response to abiotic stresses. Therefore, in order to improve the stress tolerance and yield of trees by genetic engineering, the function of a gene must be identified by transforming it into a tree genome.

The *TaLEA* is a stress tolerance gene, which has been successfully introduced into tobacco [11], and has been shown to confer tolerance against drought stress. Overexpression of *TaLEA* by transgenic tobacco has been reported to improve resistance to drought tolerance by maintaining cell membrane stability and exhibiting less reduction in plant growth under drought conditions. Our results were consistent with these findings, suggesting that the *TaLEA* gene may share the same roles, and could be involved in the same pathways under drought conditions in poplar and tobacco plants.

In addition, the current study showed that the *TaLEA* gene enhanced salt tolerance ability of transgenic poplars. Similarly, MsLEA3-1, a *LEA* protein from alfalfa (*Medicago sativa* L.), was confirmed in improving resistance to high salinity when expressed in tobacco [21]. Transgenic sweet potato non-embryogeneic calli overexpressing the IBLEA14 from sweet potato (*Ipomoea batatas*) showed enhanced tolerance to drought and salt stresses, whereas RNAi-treated calli exhibited increased stress sensitivity [22]. The ectopic expression of DHN-5, a wheat ( *Triticum* *durum* ) group 2 *LEA* protein, contributed to an improved tolerance to salt and drought stress [23].

In transgenic lines, the MDA content and the relative electrolyte leakage were lower than in non-transgenic plants, indicating that introduction of the *TaLEA* gene protected the cell membranes and decreased membrane damage under the salt stress. We deduced that a salt tolerance mechanism was conferred by the *TaLEA* gene product through protection of cell membranes from damage, and this result is consistent with the previous findings by Bai et al. [21].

Interestingly, not all transgenic lines have same salt and drought stress tolerance, such as the height growth rate and stress damage. The transgenic lines 1, 9, 11 and 14 showed higher growth rate and relatively lower stress damages under salt and drought stresses. On the other hand, the *TaLEA* gene expression levels of four transgenic lines were apparent. The expression of lines 9 and 14 shared a high level. While the lines 1 and 11 showed a moderate level of expression. These results suggest that the expression levels of exogenous resistance gene and stress resistance were not positively correlated, and that the appropriate expression of *TaLEA* gene is sufficient to improve the resistance of plants.

Among the lines analyzed for salt stress tolerance, the transgenic line 11 was the most salt-tolerant, as it showed the lowest stress damage and the highest relative height growth rate under stress conditions compared to the non-transgenic plants and other transgenic lines. It provides a good candidate line to improve salt tolerance in future studies. As for the salt tolerance mechanism of the T11 line, we speculate that the insertion site of *TaLEA* may be within a gene or its regulatory regions inhibiting stress tolerance. This could have caused changed expression and function of this gene or other related genes. In addition, another product was apparently observed in the amplification product of the T11 line with the *TaLEA* gene primer PCR. It is, therefore, essential to study the regulation mechanisms of T11 to salt and drought stress tolerance in the future.

The tolerance of salt and drought stresses is determined by the coordination of multiple gene expression. Many genes have been shown to be involved in tolerance of salt and drought stresses, such as AtSKIP [24], zinc finger protein [25], heat shock protein HSP [26] and DREB [27]. Most of these genes were a multi-gene family. Therefore, a multi-gene transformation strategy will be the main means of gene engineering for practical application. In order to improve the salt and drought tolerance of trees, cloning and characterization of other *LEA* genes in *T* *. androssowii* and other stress-related genes will be studied in the future.

## Materials and Methods

### Plant transformation and selection

The cDNA for an ORF of *TaLEA* gene was amplified from the pDNR–LIB vector and cloned into the SacI and XbaI sites of plant expression vector pROKII to generate the *TaLEA* gene driven by the CaMV 35S promoter as described by Wang et al. [11]. The leaves of genetically identical *Populus* *simonii* × *P* *. nigra* plantlets were used as explants for infection [28]. The leaves were cut and pre-cultured in MH solid medium (Murashige and Skoog medium added with 0.1 mg L^-1^ folacin and 0.1 mg L^-1^ biotin) containing 0.05 mg L^-1^ NAA and 0.5 mg L^-1^ 6-BA (named as differentiation medium) for two days. The pre-cultured explants were then infected with *Agrobacterium* in a way that the absorbance value at wavelength of 600 nm (OD_600_) was 0.1 for 2 min. The infected explants were co-cultivated in differentiation medium in the dark for 2-3 d at 25°C. After co-cultivation, explants were transferred to plates of differentiation medium with 200 mg L^-1^ cefotaxime for two days, and were then transferred to selective medium (MH + 0.05 mg L^-1^ NAA + 0.5 mg L^-1^ 6-BA + 50 mg L^-1^ kanamycin + 200 mg L^-1^ cefotaxime). After 20-30 days, the kanamycin-resistant shoots were generated on selective medium. When the adventitious shoots grew to about 3-4 cm in length, they were transferred to rooting medium (MH + 0.2 mg L^-1^ IBA + 50 mg L^-1^ kanamycin + 200 mg L^-1^ cefotaxime) to let them develop roots.

### Polymerase chain reaction (PCR) and ribonucleic acid (RNA) gel blot analysis

Total DNA was extracted from non-transgenic plants and 11 transgenic lines using a CTAB protocol. A 312 bp fragment of *TaLEA* was amplified using the 5’-atggctcgctgctcttactc-3’ (forward) and 5’-tcagtgagaggatcgattgaac-3’ (reverse) primers. The reaction mixture (20 µL) contained 2 µL of 10 × PCR buffer, 0.5 µM of each forward and reverse primer, 200 µM dNTP, 1 U Taq (Takara Bio) and 100 ng of DNA template. The amplification was completed using the following cycling parameters: 94°C for 3 min, followed by 30 cycles at 94°C for 30 s, at 58°C for 30 s and at 72°C for 40 s. The amplified fragments were electrophoresed on 1% (w/v) agarose gels, and detected using ethidium bromide along with molecular weight markers.

To investigate the expression of *TaLEA* in transgenic poplars, total RNA was isolated from transgenic lines and non-transgenic plants using the CTAB protocol. The extracted RNA was treated with DNase (RNase free) to remove DNA contamination. Twenty micrograms of total RNA were fractionated on 1% agarose-formaldehyde gels and transferred onto Hybond N^+^ membranes (Amersham Pharmacia). The probes were labeled with digoxigenin (DIG)-dUTP by PCR using open reading frame cDNA as a template. Hybridization was carried out at 68°C for 18 h. Hybridization signals were detected with CDP-Star (Tropix) using FujiFilm LAS-1000plus.

### Salt and drought treatment of transgenic poplars

Transgenic and non-transgenic poplar plants were grown in 17 cm by 21 cm pots containing a mixture of peat and sand (2: 1 v/v) under controlled greenhouse conditions of 70-75% relative humidity, 14 h light, and an average temperature of 24°C. At least five plants (plant clones from the transgenic plants) from each line were used in each experiment. The well-watered plants (about 100 cm in length) were tested for salt and drought stresses. For the drought tolerance test, water was withheld from the plants for seven days. For the salt treatment, the plants were watered directly on their roots with 200 mM NaCl solution for six days. During the treatment period, the plants were watered on the roots with NaCl solution every day. Following these treatments, plant leaves were harvested for a physiological parameter analysis.

### Measurement of malonyldialdehyde levels

The MDA content was determined with thiobarbituric acid (TBA) following the protocol of Wang et al. [11] with few modifications. Specially, after adding TBA (10%, 3 ml), the mixture was incubated at room temperature for 30 min. The absorbance of the supernatant was measured at 532 nm and 450 nm. The MDA concentration was calculated using the following formula:

MDA (μM) = 6.45OD_532_ -0.56OD_450_

### Relative electrolyte leakage assay

The transgenic lines and non-transgenic plants were treated with 200 mM NaCl for 6 d and withholding water for 7 d, respectively. The third leaf from the top of each plant was used for the electrolyte leakage assay, as described by Wang et al. [11]. The relative electrolyte leakage (REL) was calculated using the following formula:

REL = (S1/S2) × 100%.

Where, S1 indicates the electrolyte conductivity after vacuuming for 15 min, S2 indicates the electrolyte conductivity at 90°C after 20 min, and they were measured after cooling at the room temperature.

### Percentage of wilted leaves and relative rate of height growth measurement

The measurement of plant height taken before introducing salt and drought stresses was used as the basic value. The transgenic lines and non-transgenic plants were treated in a greenhouse with 200 mM NaCl and withholding water for 6 d and 7 d, respectively. After 30 d of stresses, the height (final values) and the number of wilted leaves (dead leaves or drooping and yellowing leaves) of non-transgenic plants and transgenic lines were investigated. The relative rate of height growth (RHG) was calculated as:

RHG = (final value - basic value)/basic value × 100%.

The percentage of wilted leaves was calculated by the formula:

Percentage of wilted leaves = (number of wilted leaves/total number of leaves) × 100%.

### Statistical analyses

Data were analyzed using the SPSS software package (SPSS, Chicago, USA). Tukey’s multiple comparison tests was used for data analyses in individual performances. For all analyses, the level of significance was set at *P* < 0.05. The sample variability was given as the standard deviation (S.D.).

## Conclusions

To identify the function of *TaLEA* gene in tree species under abiotic stresses, transgenic poplar lines with exogenous *TaLEA* gene were generated. Compared with the wild type (WT) poplar, some transgenic lines significantly decreased the MDA content and relative electrolyte leakage. The relative height growth rates in transgenic plants were significantly higher than that in the WT plants under salt and droughts stress conditions. The results suggested that overexpression of the *TaLEA* gene could enhance salt and drought stress resistance in forest trees by mediating some physiological processes associated with salt and/or drought tolerance of plants.
